# Correction: Smartphone-assisted optical quantification of d-penicillamine in human plasma sample using toluidine blue as efficient chromogenic probe: introducing a novel portable device toward therapeutic drug monitoring (TDM)

**DOI:** 10.1039/d6ra90163h

**Published:** 2026-11-06

**Authors:** Fatemeh Nami, Farnaz Bahavarnia, Mohammad Hasanzadeh, Nasrin Shadjou

**Affiliations:** a Nutrition Research Center, Tabriz University of Medical Sciences Tabriz Iran; b Food and Drug Safety Research Center, Pharmaceutical Sciences Institute, Tabriz University of Medical Sciences Tabriz Iran; c Pharmaceutical Analysis Research Center, Pharmaceutical Science Institute, Tabriz University of Medical Sciences Tabriz Iran hasanzadehm@tbzmed.ac.ir; d Department of Nanotechnology, Faculty of Chemistry, Urmia University Urmia Iran

## Abstract

Correction for ‘Smartphone-assisted optical quantification of d-penicillamine in human plasma sample using toluidine blue as efficient chromogenic probe: introducing a novel portable device toward therapeutic drug monitoring (TDM)’ by Fatemeh Nami *et al.*, *RSC Adv.*, 2026, **16**, 63035–63045, https://doi.org/10.1039/d6ra07917b.

The authors regret that the title of the original manuscript contained a typo. The correct title is as shown above.

The Royal Society of Chemistry apologises for these errors and any consequent inconvenience to authors and readers.

